# Effectiveness of a tailored web app on sun protection intentions and its implications for skin cancer prevention: A randomized controlled trial

**DOI:** 10.1371/journal.pdig.0000032

**Published:** 2022-05-12

**Authors:** Vasileios Nittas, Margot Mütsch, Tobias Frey, Julia Braun, Milo A. Puhan

**Affiliations:** 1 Epidemiology, Biostatistics and Prevention Institute, University of Zurich, CH-8001 Zurich, Switzerland; 2 Department of Communication and Media Research, University of Zurich, CH-8050 Zurich, Switzerland; Public Health Institute, UNITED STATES

## Abstract

Skin cancers related to sunexposure are rising globally, yet largely preventable. Digital solutions enable individually tailored prevention and may play a crucial role in reducing disease burden. We developed SUNsitive, a theory-guided web app to facilitate sun protection and skin cancer prevention. The app collected relevant information through a questionnaire and provided tailored feedback on personal risk, adequate sun protection, skin cancer prevention, and overall skin health. SUNsitive’s effect on sun protection intentions and a set of secondary outcomes was evaluated with a two-arm randomized controlled trial (n = 244). At 2 weeks post-intervention, we did not find any statistical evidence for the intervention’s effect on the primary outcome or any of the secondary outcomes. However, both groups reported improved intentions to sun protect compared to their baseline values. Furthermore, our process outcomes suggest that approaching sun protection and skin cancer prevention with a digital tailored “questionnaire–feedback” format is feasible, well-perceived, and well accepted.

**Trial registration**: Protocol registration: ISRCTN registry (ISRCTN10581468).

## Author summary

Many countries around the world, including the country we live in, Switzerland, face an increasing incidence of skin cancers, known to be partially due to excessive exposure to sunlight. In the future, incidence can be reduced if more people better protected their skin from the sun. Nonetheless, skin cancer prevention is often not adequately addressed in regular primary care or travel consultations. Here, we developed an app (SUNsitive), accessible via any internet browser that aims to support individuals protect from excessive sun exposure to prevent skin cancer. It does so in a tailored manner, adjusting its content to different groups of users. We tested SUNsitive with 244 participants, 119 of them randomly assigned to receive full access to the app and 125 just a basic non-tailored version of it, aiming to see whether the first group would have improved intentions to protect from the sun 2 weeks later. Our findings did not find any sign that the tailored intervention improved intentions, however, they show that our app was well-perceived, accepted, and considered as useful and feasible by participants. Future digital skin cancer prevention can build upon our findings.

## Introduction

Sun-exposure-related skin cancers, such as malignant melanoma or keratinocyte skin cancer are on a global rise [1–3]. With 25,000 annual cases, Switzerland is facing a steadily increasing incidence, being the country with the highest overall skin cancer burden in Europe [4]. Every tenth case is attributed to melanoma, the most dangerous and fatal type of skin cancer, primarily affecting the young and middle-aged population [3]. Most melanoma cases originate from exposure to ultraviolet (UV) light, emitted by the sun or artificial sources (e.g., solarium). Beyond non-controllable factors (i.e. genetic predisposition), much of that disease burden is preventable. Relatively simple behavioral changes, such as proper use of sunscreens and protective clothing as well as the avoidance of direct sunlight and tanning salons can mitigate a considerable part of that risk [5,6].

Although the risks are well known, sunburns remain frequently reported across many European countries, as well as the United States and Australia, especially among younger age groups [7–9]. In the case of Switzerland, regular exposure to intense UV radiation due to activities at high altitudes and related to winter sport additionally increases the overall risk for sunburns. Strategies to identify high-risk individuals for target group-oriented preventive efforts are considered key to reducing disease burden [3]. Previous research highlights the effectiveness of interventions that selectively address high-risk population groups using tailored messages to induce behavior change [10–12].

Today’s intervention efforts increasingly rely on digital devices and channels to take advantage of the fast adaption and high potential reach. Digital solutions facilitate tailoring through automatically personalized messaging, engaging interactivity, and a user-centered design. Previous applications utilized mobile devices to deliver individualized and real-time sun protection advice [13,14], or made use of digital avatars to guide and support sun-related behavior change [15]. Nonetheless, randomized controlled trials that evaluated the effects of technology-mediated promotion of sun protection and prevention of skin cancer remain scarce. Our study aims to be a step towards filling that gap.

SUNsitive is a stand-alone, web-based application (app), developed to provide tailored feedback on skin cancer prevention through UV-light protection (sun, solarium). It consists of a questionnaire part, where users enter relevant sociodemographic and health behavior-related information, and a feedback part, where users receive individually tailored information on skin health and skin cancer prevention, skin appearance (UV-light induced premature aging) and other skin-damaging health behaviors, including smoking. The tailored feedback is dependent on the questionnaire answers of each user. The web apps design is based on principles of (1) tailoring, (2) interactivity, and (3) user-centeredness. Tailoring ensures that any provided information is adapted to the receiving individual and therefore increases its relevance and impact [16]. Interactivity ensures that information exchange is neither passive nor one-sided, facilitating active participation. User-centeredness ensures that the app’s design and content are user-friendly, developed with and for users.

### Objectives

Our study aims to evaluate SUNsitive’s effect on sun protection intentions (primary outcome), as well as a set of secondary outcomes, including sun protection self-efficacy, attitudes towards tanning, solarium use intentions, and smoking status. We hypothesized that exposure to SUNsitive would improve short-term sun protection intentions, as well as related self-efficacy and attitudes.

## Methods

### Study design

We conducted a randomized controlled trial with 2 arms and a 2-week follow-up period. Participants of the intervention arm received full access to the SUNsitive app. That included (a) the baseline questionnaire, followed by (b) the tailored risk and educational feedback. The participants of the study’s control arm received limited access to the SUNsitive app. That included (a) the baseline questionnaire, followed by (a) a brief, generic (non-tailored) information on the risks of sun exposure. A protocol was registered with the ISRCTN registry (ISRCTN10581468) [17].

### App development and piloting

SUNsitive’s content was theory-guided, combining Aijzen’s theory of planned behavior., Banduras social cognitive theory, as well as DiClemenente and Prochaska’s transtheoretical model of behavior change [18–20]. The theory of planned behavior aims to predict human behavior through intentions, which in turn are influenced by attitudes, normative beliefs, and perceived control. The social cognitive theory explains knowledge acquisition through social and external forces, including social interactions, while the transtheoretical model of behavior change explains change in human behavior through different stages of readiness, each requiring a different set of behavior change techniques. Combining these three theories, we targeted behavior constructs such as attitudes, intentions, and self-efficacy, using behavior change communication approaches such as persuasion, motivation, and skill enhancement. The SUNsitive app is embedded within an already existing digital online platform of the Epidemiology, Biostatistics and Prevention Institute of the University of Zurich, the research management information system (RMIS).

The first version of SUNsitive was piloted (n = 10) qualitatively using the think-aloud methodology. Participants were identified through a research participant list of the University of Zurich Travel Clinic and invited to 30-minute-long online interviews via E-Mail. Interested participants were asked to register through the application’s website, fill out the baseline questionnaire, and read all feedback modules. Additionally, they were asked to simultaneously express their thoughts and impressions aloud. Information was maximized with follow-up questions. All think-aloud interviews were conducted and recorded via ZOOM. Interviewees provided informed consent and agreed to be recorded. The pilot’s results were used to improve SUNsitive’s structure and content in terms of user-friendliness and clarity. That included language adjustments, revisions of module lengths, and the restructuring of the provided information.

### Participants and recruitment

Recruitment was conducted by LINK Marketing Services AG, a Swiss market and social research institute. Participants were recruited online, through LINK’s extensive population-based online panel. Based on predefined age (equal number of females and males) and sex quotas (equal number in all age groups 18–29; 30–44; 45–59; 60–75), and expecting a participation rate of about 10%, a random sample of about 3,000 participants was drawn from the larger panel and invited to participate via E-Mail. The invitation included detailed information on the study’s aims, eligibility criteria, as well as a direct link to the SUNsitive’s registration webpage. Eligible participants had to be of 18 years of age or above, able to speak German and provide independent informed consent, have no history of skin cancer, and be residents in Switzerland.

Out of the 3000 invited, only those fulfilling all eligibility criteria and accepting participation were randomly allocated to either the intervention or the control group. Randomization was computerized and neither Link nor the research team had access or any influence over it, i.e. ensuring concealment of random allocation. Baseline recruitment was conducted between the 20^th^ of November 2020 and the 3^rd^ of December 2020. Each pilot participant received a 50 CHF grocery voucher. After the pilot, each trial participant received a 2 CHF credit for initial participation (baseline questionnaire and intervention) and a further 30 CHF for completing the follow-up questionnaire (a total of 32 CHF for each trial participant).

### Data collection

#### Baseline questionnaire

Upon acceptance and randomization, participants were directed to the SUNsitive web app. After successful registration, participants were asked to confirm their E-Mail addresses, upon which automatic user profiles with anonymized user IDs were generated. During first log-in, users were automatically directed to the study’s electronic informed consent interface, providing detailed information on the study’s aims and processes, potential risks, participant rights, as well as privacy and data security. Participants that provided informed consent were instructed to fill out the baseline questionnaire, entailing questions on sociodemographics (sex, age, civil status, income, education), sun protection intentions, sun protection self-efficacy, attitudes towards tanning, sun exposure at high altitudes, solarium use intentions and smoking status (described in detail in the outcomes section).

#### Intervention

After completion of the baseline questionnaires, participants in the intervention group received full access to SUNsitive’s feedback modules. Based on the questionnaire data, each user received tailored risk and educational information. This information is structured in thematic modules of text and graphics. While each module addresses a single topic, overall, users receive holistic feedback on their health behavior, potential risks, as well as ways to prevent skin cancer and premature aging. All modules are tailored to each participant’s reported health behavior, as reported during the baseline questionnaire, avoiding unnecessary and redundant information. For example, participants who indicated frequent UV exposure at high altitudes or solariums visits received extended modules on the associated risks and their impact on skin appearance and health. In a second example, participants who indicated positive attitudes towards sunbathing received extended information on the risks of sun exposure on skin health and aging. Those with negative (healthy) attitudes towards sunbathing received a module with a motivational statement and concise risk information. We did not use sociodemographic data for tailoring. We did not tailor according to socio-demographics as that would require strong assumptions that might not necessarily be met.

Module 1 addressed the health dangers of sun exposure, with an emphasis on the consequences of tanning and sunburns, as well as melanoma risk. Module 2 outlined the effects of sunlight on skin’s appearance, focusing on premature aging and providing visual summaries of the photoaging processes. This was accompanied by pictures of a person’s face after 5 and 10 years of sun exposure, with and without adequate sun protection, generated with the sunface app [21]. Module 3 addressed the topic of sun protection, providing a summary of all key protection measures (shade, clothing, sunglasses, sunscreen) and associated mishaps. The emphasis was placed on the inadequacy of sunscreen as a single protection measure. Module 4 listed some key myths around sun protection, with short explanations on why they do not hold. Module 5 provided a visual guide on how to self-check the skin for potentially malignant lesions or other changes, as well as advice on when to best contact a dermatologist. Module 6 summarized the risks of sun exposure at higher altitudes, providing relatable examples of Swiss mountains, while module 7 outlined the damaging effects of artificial tanning (solarium) on skin health and appearance. Finally, module 8 summarized the effects of tobacco smoking on the skin’s health, focusing primarily on appearance. As with UV light, smoking has a damaging effect on the skin, facilitating premature aging. The module’s emphasis was placed on premature aging, with pictures showing a young person’s face after three and 6 years of smoking a pack of cigarettes a day, versus not smoking at all, generated with a smokerface app [22]. The module structure allows users to choose themes and topics of highest interest, avoiding an overload of information at once.

#### Follow-up

Two weeks after initial participation, all registered participants received a second invitation to log in to SUNsitive, re-access their feedback modules, and complete the follow-up questionnaires. These were identical to the baseline questionnaires (primary and secondary outcomes), however, excluding the sociodemographics section and adding questions regarding process outcomes.

### Outcomes

#### Primary Outcomes

Our primary outcome consisted of sun protection intentions, measured by a 16-item questionnaire, adapted from Mahler et al. [23]. The questionnaire measured intentions to protect from the sun in the future (e.g. next sunny holiday or upcoming summer), focusing on the use of sunscreen (e.g. I plan to use sunscreen regularly) and protective clothing (e.g. I plan to wear shirts with long sleeves when I have to be outdoors between 10 am and 2 pm), the limitation of outdoor activities during peak sunshine hours (e.g. I plan to avoid being outdoors between the hours of 10 am and 2 pm whenever possible) and the active seeking of shade (e.g. I plan to seek out shady areas when I have to be outdoors). Participants had to rate their intentions on a 5-point Likert scale (1 = strongly disagree, 5 = strongly agree). The final score consisted of the average of all items. Cronbach’s alpha (α) was calculated to quantify the reliability of that averaged score in providing an accurate summary of all items (>0.7 = acceptable, >0.8 = good, .0.9 = excellent) [24]. The Cronbach’s alpha for our primary outcome was α = 0.88, indicating good reliability.

#### Secondary outcomes

Our secondary outcomes consisted of (a) sun protection self-efficacy, (b) attitudes towards tanning, (c) solarium use intentions, and (d) smoking status. Sun protection self-efficacy was measured by a 6-item questionnaire (e.g. how confident are you that you would stay in the shade while all your friends are enjoying themselves in the sun) adapted from Babbin and colleagues [25] and measured with a 5-point Likert scale (1 = not all confident, 5 = extremely confident). Attitudes towards tanning were measured by a 6-item questionnaire (e.g. having a tan makes me look healthy) adapted from Mahler et al. [23] and measured with a 5-point Likert scale (1 = strongly disagree, 5 = strongly agree). Solarium use intentions were measured by a 2-item questionnaire (e.g. are you planning to use a solarium sometime in the future) adapted from Heckman et al. and measured by a 5-point Likert scale (1 = definitely not, 5 = definitely yes) [26]. Finally, smoking status was measured with a state of change questionnaire adapted from Etter et al. [27]. Summarized scores were calculated for sun protection self-efficacy (α = 0.71), attitudes towards tanning (α = 0.88), and solarium use intentions (α = 0.73). We additionally asked participants whether they spend a considerable time at higher altitudes and mountain areas.

#### Process outcomes

To better understand SUNsitive’s feasibility and usability, we asked intervention participants, to provide their feedback on the clarity of questionnaires and feedback modules, the perceived relevance, trustworthiness, and motivational influence of provided information, as well as the user-friendliness and design of the web app. All process outcomes were measured at follow-up and were not adapted from a standardized scale but developed by the research team to best capture the study’s needs. We included 12 Likert Scale questions (1 = strongly disagree, 5 = strongly agree), for example, "the questionnaire was clear and easy to understand”, "the feedback was personally relevant”, “the feedback motivated me to keep protecting my skin or protect it more than I used to”. To gain a deeper understanding of user experience, we also included eight open-ended questions, such as “which feedback elements were most/least interesting?” or “which feedback elements were unclear or difficult to understand?”.

### Statistical analysis

The sociodemographic data of the two groups were explored descriptively (means, standard deviations, and range for continuous variables, numbers, and percentages for categorical variables). Additionally, we used the Mann-Whitney test to retrieve unadjusted mean score changes, reporting mean (SD) for each group (intervention, n = 119; control, n = 125), associated *p-*values, as well as between-group differences with 95% confidence intervals. Wilcoxon tests were used to explore within-group baseline and follow-up scores, reporting before and after means (SD) and associated *p-* values.

SUNsitive’s effectiveness was evaluated by comparing between-group changes in intentions to sun protect (primary outcome) and all secondary outcomes (sun protection self-efficacy, attitudes towards tanning, solarium use intentions, and smoking status). As each outcome was composed of multiple items, we calculated individual mean scores. We started with complete case analysis, comparing mean follow-up scores using linear regression and controlling for mean baseline scores and sex, due to a small imbalance. Each outcome was analyzed in a separate regression model. The results of our five analyses are reported as β coefficients, associated standard errors, confidence intervals, and *p-*values. We used multiple imputations (10 imputed datasets) to assess the potential effect of follow-up losses on our results as well as the overall robustness of our complete case analyses coefficients. The sample size was calculated for an effect size of 0.5 (primary outcome), with a power of 0.9 and significance level of 0.05, yielding 85 participants per arm, thus a minimum of 170 participants in total. All analyses were performed in R (version 4.1.1).

### Ethics approval

An ethics approval was requested by the Cantonal Ethics Committee Zurich and was waived as the research project did not fall within the scope of the Human Research Act (HRA).

## Results

**Fig 1:**
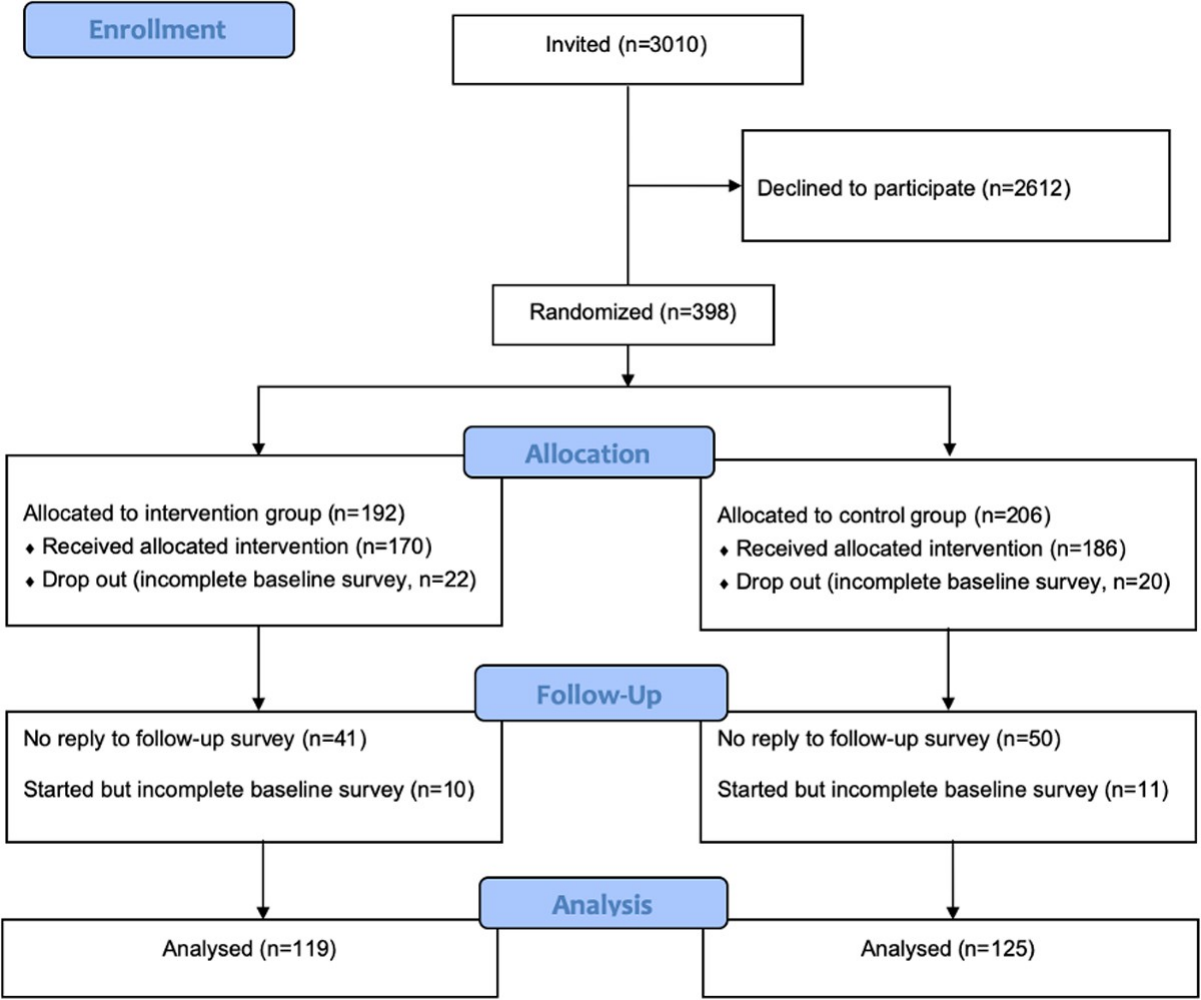
CONSORT Flow Diagram.

The full recruitment and study inclusion process is shown in the CONSORT flow diagram of Fig 1 [28]. Invitations were sent to 3010 individuals, to which 398 (13%) replied and were subsequently randomized. The baseline questionnaire was completed by 356 participants. Out of these, 244 completed the follow-up questionnaire and were included in the final analysis. The intervention arm consisted of 119, and the control of 125 participants. There were no sociodemographic differences between those lost to follow-up and those competing in the final questionnaire.

### Participant characteristics

Overall, there were no relevant sociodemographic differences between study groups in terms of age, marital status, income, and education. There was a small potentially relevant imbalance in the sex distribution of both groups. The sociodemographic distribution of both groups is provided in Table 1.

**Table 1:** Demographics of participants in the randomized controlled trial by condition.

	Intervention (N = 119)	Control (N = 125)
Mean age, years (range)	47.6 (18–78)	47.8 (19–77)
**Sex, n (%)**		
Male	65 (54.6)	60 (48.0)
Female	54 (45.4)	64 (51.2)
Other	0 (0)	1 (0.8)
**Marital Status, n (%)**		
Single	33 (27.7)	42 (33.6)
Married	67 (56.3)	62 (49.6)
Divorced	12 (10.1)	15 (12)
Registered partnership	2 (1.7)	1 (0.8)
Dissolved partnership	2 (1.7)	2 (1.6)
Widowed	3 (2.5)	3 (2.4)
**Gross Annual Income, n (%)**		
< 60.000 CHF	31 (26.1)	45 (36.0)
60.000–90.000 CHF	41 (34.4)	38 (30.4)
90.000–120.000 CHF	25 (21)	28 (20.0)
120.000–150.000 CHF	8 (6.7)	7 (5.6)
> 150.000 CHF	5 (4.2)	1 (0.8)
NA	9 (7.6)	6 (4.8)
**Education, n (%)**		
No school diploma	1 (0.8)	0 (0.0)
Lower secondary education	5 (4.2)	17 (13.6)
Upper secondary education	46 (38.7)	42 (33.6)
Tertiary education	67 (56.3)	66 (52.8)

### Intentions, self-efficacy, attitudes, and smoking status

Table 2 provides a between-group comparison of mean primary and secondary outcome scores at follow-up. No significant difference between the two arms was found.

Our within-group analyses indicate that both groups had improved intentions to sun protect compared to their baseline values (*p* = < .001). Intervention group participants additionally had improved sun protection self-efficacy (*p* = .007) and solarium use intentions scores (*p* = .012), again compared to their baseline values. Control group participants had improved healthy attitudes towards tanning (*p* = .042). A within-group comparison between baseline and follow-up values across all outcomes is available in S1 Table.

**Table 2:** Mean primary and secondary outcome scores at follow-up (Mann-Whitney tests).

Outcome	Control Mean, SD	Intervention Mean, SD	Mean Difference [95% CI]	*p*
Sun protection intentions	2.64 ± 0.6	2.73 ± 0.6	- 0.09 [-0.25, 0.07]	.25
Sun protection self-efficacy	2.29 ± 0.7	2.40 ± 0.7	- 0.11 [-0.29, 0.07]	.23
Attitudes towards tanning	1.96 ± 0.9	2.00 ± 0.8	- 0.04 [-0.25, 0.18]	.68
Solarium use intentions	3.53 ± 0.8	3.58 ± 0.7	- 0.05 [-0.25, 0.14]	.69
Smoking status	3.82 ± 1.7	4.03 ± 1.5	- 0.21 [-0.62, 0.2]	.28

Based on the results of our complete case regression analysis and adjusting for sex, we did not find any statistical evidence for the intervention’s effect on the primary outcome or any of the secondary outcomes. But we nevertheless found that the direction of effects slightly favored the intervention group for sun protection intentions as well as for most secondary outcomes, i.e. sun protection self-efficacy, attitudes towards tanning, and intentions to use the solarium. The detailed results of our complete case regression analyses are reported in S2 Table. Our multiple imputation analyses, accounting for all randomized participants that completed the baseline questionnaires but were lost to follow-up provided comparable results.

### Process outcomes

About 98% (n = 117) of intervention participants and 97% (n = 121) of control participants considered the SUNsitive’s first part, the questionnaire, to be clear and very understandable, while only 2% (n = 6) and 3% (n = 4) respectively were undecided or disagreed. The second part, the feedback, was considered clear and understandable by 96% (n = 115) of intervention participants and 87% (n = 109) of control participants, while the remaining 4% (n = 4) and 13% (n = 16) respectively were undecided. About 69% (n = 82) of intervention participants, who received full feedback considered the modules to be of adequate length, 22% (n = 26) were undecided, while 9% (n = 11) wished for more concise content. About 76% (n = 96) of control participants, who received a generic, short feedback considered it to be of adequate length, while 9% (n = 11) provided were undecided and 3% considered it to be too long. Most intervention participants (89%, n = 106) considered the provided information to be trustworthy, 9% (n = 11) were undecided, while 2% (n = 3) disagreed. In the control group, 78% (n = 98) perceived the feedback as trustworthy, 14% (n = 17) were undecided and 6% (n = 8) disagreed.

The feedback was considered personally relevant by 75% (n = 90) of the intervention group, while 14% (n = 17) were undecided and 11% (n = 12) did not consider all feedback elements relevant to their current situation. Amongst control group participants, 64% (n = 80) considered the short, generic feedback relevant, while 22% (n = 27) were undecided and 2% (n = 3) disagreed. The long-term health damage of UV-radiation, as well as the sunscreen use facts, were perceived as the most relevant feedback elements, followed by general sun protection advice and appearance-based feedback (aging). Conversely, the effects of smoking on skin health and appearance, the damaging effects of solarium use, and the use of protective clothing were considered the least relevant feedback elements.

Overall, 83% (n = 99) of intervention participants considered the feedback content to be interesting, while 13% (n = 16%) were neutral and 4% (n = 4) disagreed. About 76% (n = 95) of control group participants considered the feedback interesting, 17% (n = 21) were undecided and 7% (n = 9) disagreed. Again, the long-term UV-light-related health damage, the sunscreen use facts, and aging were considered most interesting. Although solarium use and sun-protective clothing were not considered very relevant by many, they scored high in terms of interest, potentially adding new information for those who do not smoke or have ever visited a solarium.

Most participants in the intervention group (48%, n = 56) agreed that SUNsitive’s feedback improved their knowledge of protection the risk of skin cancer, while 23% (n = 27) were undecided and 30% (n = 36) disagreed. About 41% (n = 51) of control group participants agreed that SUNsitive’s feedback improved their knowledge of sun protection and skin cancer prevention, 25% (n = 31) and 36% (n = 43) disagreed. About 76% (n = 91) of intervention group participants considered SUNsitive to have motivated them to keep protecting their skin, or even improve their sun-protective behavior, while 15% (n = 18) were undecided and 9% (n = 10) disagreed. For control group participants, 67% (n = 63) considered SUNsitive to have motivated them to keep protecting their skin, or even improve their sun-protective behavior, 17% (n = 21) were undecided and 15% (n = 20) disagreed. All process outcomes are summarized in Table 3.

**Table 3:** Process outcomes in the intervention and control group.

	Intervention n (%)		Control (n = 125) n (%)	
**The questionnaire was clear and easy to understand**				
I fully agree	76	(64)	84	(67)
I agree	41	(34)	37	(30)
I am not sure	1	(1)	2	(1.5)
I disagree	1	(1)	2	(1.5)
I fully disagree	0	(0)	0	(0)
Not answered	0	(0)	0	(0)
**The feedback was personally relevant**				
I fully agree	16	(13)	22	(18)
I agree	74	(62)	58	(46)
I am not sure	17	(14)	27	(22)
I disagree	12	(11)	14	(11)
I fully disagree	0	(0)	3	(2)
Not answered	0	(0)	1	(1)
**The feedback was interesting**				
I fully agree	23	(19)	24	(19)
I agree	76	(64)	71	(57)
I am not sure	16	(13)	21	(17)
I disagree	4	(4)	8	(6)
I fully disagree	0	(0)	1	(1)
Not answered	0	(0)	0	(0)
**The feedback improved my knowledge of skin cancer and sun protection**				
I fully agree	11	(9)	10	(8)
I agree	45	(38)	41	(33)]
I am not sure	27	(23)	31	(25)
I disagree	30	(25)	34	(27)
I fully disagree	6	(5)	9	(7)
Not answered	0	(0)	0	(0)
**The feedback was clear and easy to understand**				
I fully agree	42	(35)	49	(39)
I agree	73	(61)	60	(48)
I am not sure	4	(4)	16	(13)
I disagree	0	(0)	0	(0)
I fully disagree	0	(0)	0	(0)
Not answered	0	(0)	0	(0)
**The feedback was too long**				
I fully agree	1	(1)	2	(2)
I agree	10	(8)	1	(1)
I am not sure	26	(22)	11	(9)
I disagree	69	(58)	83	(66)
I fully disagree	10	(8)	13	(10)
Not answered	3	(3)	15	(12)
**The feedback was trustworthy**				
I fully agree	30	(25)	29	(23)
I agree	76	(64)	69	(55)
I am not sure	11	(9)	17	(14)
I disagree	1	(1)	5	(4)
I fully disagree	2	(1)	3	(2)
Not answered	0	(0)	2	(2)
**The feedback motivated me to keep protecting my skin or protect it more than I used to**				
I fully agree	25	(21)	26	(21)
I agree	66	(55)	57	(4)
I am not sure	18	(15)	21	(17)
I disagree	9	(8)	17	(13)
I fully disagree	1	(1)	3	(2)
Not answered	0	(0)	1	(1)

## Discussion

### Principal findings

SUNsitive was developed to provide a stand-alone, individually tailored feedback on skin cancer prevention through UV-light protection (sun, solarium), ultimately allowing for a comprehensive preventive consultation outside the clinical setting. Its design and content were theory-guided and based on tailoring, interactivity, and user-centeredness. Despite its careful design and extensive piloting, our trial did not provide statistical evidence for SUNsitive’s effect on primary (sun protection intentions) or secondary outcomes (sun protection self-efficacy, attitudes towards tanning, solarium use intentions, and smoking status). Nonetheless, both groups showed improvements in intentions to protect from the sun compared to their baseline. Process outcomes suggest that SUNsitive was well-perceived, and considered relevant, interesting as well as user-friendly, suggesting good acceptability and feasibility. Most participants reported that SUNsitive motivated them to keep protecting their skin or improve their protective behavior, while most agreed that SUNsitive improved their knowledge of the risks of UV radiation.

### Questionnaire, tailoring, and setting

SUNsitive’s results could be potentially explained in two ways. First, we decided to use automatic tailoring instead of a more complicated system, where users would manually adjust SUNsitive’s content to their preferences. That has its advantages and disadvantages. On one side, automatic tailoring allows for simpler and faster completion, without placing an additional decision burden on users. On the other side, automation might inevitably miss the needs and preferences of some users. Similarly, our tailoring approach did not entirely disable any of the feedback modules but instead adjusted the level and detail of information participants would receive on each topic, based on individual questionnaire answers. For example, instead of entirely disabling the module on solarium for those users who never use the solarium, we decided to show them the module with only a minimum of information. That was likely perceived as irrelevant by some users, which in turn might have reduced the overall feedback impact. The fact that at follow-up both groups showed significantly improved intentions to sun protect compared to their baseline values could indicate that our baseline questionnaire (received by both groups) triggered some cognitive processes that led to positive change. In contrast, the tailored feedback (provided only to intervention participants) might not have added much to the questionnaire’s value. Future research should investigate the value of comprehensive, yet easy-to-understand questionnaires in facilitating behavior change, or at least being willing to change.

Second, the intervention was entirely digital, deviating from its originally planned setting due to the SARS-CoV-2 pandemic. Initially, SUNsitive was designed to act as a digital prevention tool in the waiting room of a travel clinic. Participants would complete the modules while waiting for their consultation and SUNsitive’s content would then be briefly discussed with a healthcare professional, who would follow-up, clarify and re-emphasize the importance of adequate UV-light protection. Due to SARS-CoV-2, recruitment was instead conducted entirely online and without a subsequent healthcare consultation, reducing overall intervention exposure. Evidence from other fields, such as diabetes suggests that digital interventions combining multiple behavior change techniques and components tend to be more effective than those that do not [29]. Finally, SARS-CoV-2 restrictions were at high levels during SUNsitive’s implementation, affecting outdoor leisure and travel, as well as potentially our participants’ attitudes towards sun exposure.

### Acceptability and feasibility

Our process outcomes suggest that approaching sun protection and skin cancer prevention digitally, with a simple but tailored “questionnaire–feedback” format is feasible, well-perceived, and well accepted. Almost all participants considered SUNsitive’s digital preventive consultation to be clear, understandable, and trustworthy. This is attributed to SUNsitive’s interactivity, simple language, good balance between text, illustrations, and pictures, as well as its theory-guided structure.

As expected, SUNsitive’s aging-related feedback element was considered relevant and interesting by many participants. This is in line with previous research, which confirms the added value of appearance-based constructs in sun protection advice and skin cancer prevention. A randomized controlled trial by Mahler and colleagues assessed the effects of photographs and photoaging information on long-term sun protection behaviors [30]. At 12 months the interventions showed a positive effect on sun protection intentions, as well as actual behavior [30]. Similar conclusions were provided by William’s and colleagues, summarizing the 21 studies and reporting that appearance-based interventions had a positive impact on exposure and intentions, playing a potentially vital role in the prevention and health promotion [10]. While about 10% of SUNsitive’s feedback was dedicated to premature aging, an even stronger focus on appearance might have improved the app’s overall effect.

On contrary, the effects of smoking on skin health and appearance, the damaging effects of solarium use, and the use of protective clothing were considered the least relevant feedback elements. The first two probably because most participants reported neither smoking nor solariums visits. The third is probably because of its difficulty, as protective clothing is directly associated with discomfort for many.

### Limitations

Our study needs to be viewed in consideration of the following limitations. Due to the SARS-CoV-2 pandemic, the study’s implementation plan had to be changed from a hybrid approach (digital prevention in the waiting room of a travel clinic followed by an in-person consultation) to a fully digital one. While that might have had an impact on SUNsitive’s overall effect, the app and all study procedures were carefully adjusted to seamlessly operate fully online. Consequentially, participants were recruited online, through an external social research service provider, which did not allow us to directly contact participants. That led to eliminating the originally planned reminders to re-access SUNsitive one week after initial participation. Due to the app’s strict privacy features, we could not assess app engagement, which would have improved our understanding of the study’s findings. Not having app engagement data does not allow us to compare which of the two groups spend more time within the app, neither what content had the higher, or lowest reach. In fact, our findings could potentially be explained by higher engagement of control group participants (generic feedback) and lower engagement of intervention group participants (tailored feedback).

Our sample included only German-speaking and predominantly highly educated participants and might not be generalizable to the entire Swiss population. Finally, our rather short follow-up periods of two weeks might have not been adequate for measuring SUNsitive’s impact. Despite these limitations, this is one of the few randomized controlled trials in the field of digital skin cancer prevention. It has been thoroughly designed and carefully implemented, adding important knowledge to the field of digital prevention.

### Future research

Considering this study’s findings and shortcomings we believe that future research on digital skin cancer prevention should focus on (1) refined tailoring, (2) longer follow-up periods, including information pre-and post-travel, (3) the assessment of additional functions, including reminders and (4) user engagement with different app functions, as well as the content.

## Conclusion

We did not find any statistical evidence to support SUNsitive’s positive effect on sun protection intentions or any of the study’s secondary outcomes. Nonetheless, our findings support that web-based, stand-alone, and tailored skin cancer prevention approaches are feasible and seem to be overall well-perceived and accepted among internet users, with most participants reporting increased motivation to continue protecting their skin. Future research should evaluate the impact of more flexible tailoring approaches and longer intervention periods and the role of well-structured, comprehensive yet simple questionnaires in facilitating positive behavior change.

## Supporting information

S1 Consort Checklist CONSORT 2010 checklist of information to include when reporting a randomised trial. (DOC)

S1 Table Within-group comparison of mean primary and secondary outcomes (Wilcoxon Tests). (DOCX)

S2 Table Results of Complete Case Regression Analyses for primary and all secondary outcomes. (DOCX)

S1 Study Protocol Study Protocol. (DOCX)

S1 Ethics Approval Ethics approval and clarification of responsibility. (PDF)

S1 Data SUNsitive Dataset. (XLSX)
