# Supplementary material for: Effectiveness of a tailored web app on sun protection intentions and its implications for skin cancer prevention: A randomized controlled trial
Source: PLOS Digit Health. 2022 May 12;1(5):e0000032. doi: 10.1371/journal.pdig.0000032 (PMC9931317; doi:10.1371/journal.pdig.0000032)
Supplement: S1 Table — (DOCX) [file pdig.0000032.s002.docx]

## S1 Table

Within-group comparison of mean primary and secondary outcomes (Wilcoxon Tests)

| **Outcome** | **Intervention Group** | | | **Control Group** | | |
| --- | --- | --- | --- | --- | --- | --- |
|  | before  mean, SD | after  mean, SD | *P* | before  mean, SD | after  mean, SD | *P* |
| Sun protection intentions | 2.54 ± 0.6 | 2.73 ± 0.6 | <.001 | 2.48 ± 0.6 | 2.64 ± 0.6 | <.001 |
| Sun protection self-efficacy | 2.28 ± 0.7 | 2.40 ± 0.7 | .007 | 2.23 ± 0.7 | 2.29 ± 0.7 | .209 |
| Attitudes towards tanning | 2.00 ± 0.8 | 2.00 ± 0.8 | .505 | 2.04 ± 0.8 | 1.96 ± 0.9 | .042 |
| Solarium use intentions | 3.46 ± 0.9 | 3.58 ± 0.7 | .012 | 3.50 ± 0.8 | 3.53 ± 0.8 | .711 |
| Smoking status | 4.03 ± 1.6 | 4.03 ± 1.5 | .969 | 3.90 ± 1.6 | 3.82 ± 1.7 | .114 |

SD= standard deviation
