# Supplementary material for: Effectiveness of a tailored web app on sun protection intentions and its implications for skin cancer prevention: A randomized controlled trial
Source: PLOS Digit Health. 2022 May 12;1(5):e0000032. doi: 10.1371/journal.pdig.0000032 (PMC9931317; doi:10.1371/journal.pdig.0000032)
Supplement: S2 Table — (DOCX) [file pdig.0000032.s003.docx]

## S2 Table

Results of Complete Case Regression Analyses for primary and all secondary outcomes

| **Model** | **Outcome** | **β** | **SE** | **95% CI** | ***P*** |
| --- | --- | --- | --- | --- | --- |
| 1 | (Constant)  **Sun protection intentions (primary)**  Sex  Baseline intentions | 0.564  -0.051  0.030  0.833 | 0.108  0.049  0.049  0.042 | 0.386 - 0.741  -0.133 - 0.030  -0.052 - 0.111  0.764 - 0.902 | .000  .299  .546  .000 |
| 2 | (Constant)  **Sun protection self-efficacy (secondary)**  Sex  Baseline self-efficacy | 0.538  0.077  0.161  0.750 | 0.105  0.059  0.058  0.042 | 0.366 - 0.711  -0.020 - 0.174  0.065 - 0.257  0.680 - 0.820 | .000  .190  .006  .000 |
| 3 | (Constant)  **Attitudes towards tanning (secondary)**  Sex  Baseline attitudes | 0.159  0.071  -0.008  0.882 | 0.092  0.061  0.060  0.038 | 0.008 - 0.311  -0.030 - 0.172  -0.107 - 0.092  0.820 - 0.945 | .084  .246  .900  .000 |
| 4 | (Constant)  **Solarium use intentions (secondary)**  Sex  Baseline intentions | 0.891  0.086  0.013  0.750 | 0.122  0.055  0.054  0.032 | 0.690 - 1.092  -0.005 - 0.176  -0.076 - 0.102  0.697 - 0.803 | .000  .122  .808  .000 |
| 5 | (Constant)  **Smoking status (secondary)**  Sex  Baseline smoking status | 0.415  0.102  0.136  0.854 | 0.155  0.108  0.111  0.035 | 0.160 - 0.671  -0.077 - 0.281  -0.047 - 0.318  0.797 - 0.911 | .008  .348  .220  .000 |

β = regression coefficient; SE= standard error; CI= confidence interval
