# Supplementary material for: Effectiveness of a tailored web app on sun protection intentions and its implications for skin cancer prevention: A randomized controlled trial
Source: PLOS Digit Health. 2022 May 12;1(5):e0000032. doi: 10.1371/journal.pdig.0000032 (PMC9931317; doi:10.1371/journal.pdig.0000032)
Supplement: S1 Study Protocol — (DOCX) [file pdig.0000032.s004.docx]

S4 Study Protocol

ISRCTN10581468 <https://doi.org/10.1186/ISRCTN10581468>

Can technology help us improve sun protection and skin health? The SUNsitive project

**Condition category**

Cancer

**Date applied**

27/02/2020

**Date assigned**

09/03/2020

**Last edited**

20/11/2020

**Prospective/Retrospective**

Prospectively registered

**Overall trial status**

Completed

**Recruitment status**

No longer recruiting

**Plain English Summary**

Current plain English summary as of 30/09/2020: 

Background and study aims 
SUNsitive is an online application (app) that allows user to enter information relevant for their skin health (through an online questionnaire) and then uses that information to give a tailored online feedback on melanoma risk, overall skin health and skin appearance (including tobacco use effects) and advice on reducing any form of skin damage. 

The main aim of the SUNsitive study is to explore whether the app can improve people's intentions to protect themselves from the sun. It additionally aims to see whether it improves people's self-belief in protecting themselves, their beliefs and attitudes about tanning (indoor and outdoor), and their smoking status. Furthermore, SUNsitive aims to explore the experiences and satisfaction of users with the app. 

Who can participate?
German speakers Swiss residents who are aged 18 years or older, provide consent to the study and have no current or previous skin cancer.

What does the study involve?
Participants will be recruited through a market and social research company (Link Institute) and drawn from a large online panel that is representative of the Swiss population. The invitation to the study, as well as study participation, happen entirely online. Those who are eligible and
accept to participate will access the SUNsitive web app through an invitation link. Once on the app, participants will be asked to register and provide an online consent.
Each participant will be allocated to one of two groups, by chance. The first group will have full access to the app (including the online questionnaire and detailed online feedback). The second group will only have access to the online questionnaire and receive a very brief and short online feedback. For the first group, the online feedback will be tailored advice about skin health. The feedback will be based on the answers provided by the participant in the questionnaire. For the second group, the brief online feedback will be the same for everyone. The app can be accessed via any internet-connected device from anywhere each participant wants.
One week later, participants in the full access group will receive a reminder email, asking them to access the SUNsitive app from home and to read the advice and feedback given on the app again. Two weeks after the first participation round is completed, both groups will again receive an
invitation to access the app. After log-in, they will be asked to answer the initial questionnaire again, in order to see whether any changes occurred. They will also be asked to answer a short questionnaire on what they liked or disliked about SUNsitive.

What are the possible benefits and risks of participating?
SUNsitive will contribute towards a much needed and targeted increase in awareness around sun protection, melanoma prevention, skin health, and the linkages between tobacco consummation and the skin. Based on previous research, it is expected that SUNsitive exposure will impact individual attitudes towards tanning and UV-ray exposing behavior, which is a strong predictor for actual behavior.
This trial can be categorized involves minimal risk or burden. SUNsitive is non-invasive, does not target a vulnerable population, and is not expected to cause any discomfort or burden. Providing information on risks and dangers (which SUNsitive will do through its feedback modules) always carries a risk to cause individual concern or worry. SUN-sitive will counteract that by using clear, non-emotional language, based on facts and evidence. Part of the preventive feedback will be an estimation of melanoma risk, based on a validated score. We will ensure that the participants are informed that this risk score is only estimated and that it strongly depends on the accuracy of the provided information.

Where is the study run from?
Epidemiology, Biostatistics and Prevention Institute, University of Zurich (Switzerland)

When is the study starting and how long is it expected to run for?
March 2019 to January 2021

Who is funding the study?
The Béatrice Ederer-Weber Foundation (Switzerland) and the University of Zurich Digital Society Initiative (Switzerland)

Who is the main contact?
Mr Vasileios Nittas
vasileios.nittas@uzh.ch

_____

Previous plain English summary:

Background and study aims 
SUNsitive is an online application (app) that allows user to enter information relevant for their skin health (through an online questionnaire) and then uses that information to give a tailored online feedback on melanoma risk, overall skin health and skin appearance (including tobacco use effects) and advice on reducing any form of skin damage. 

The main aim of the SUNsitive study is to explore whether the app can improve people's intentions to protect themselves from the sun. It additionally aims to see whether it improves people's self-belief in protecting themselves, their beliefs and attitudes about tanning (indoor and outdoor), and their smoking status. Furthermore, SUNsitive aims to explore the experiences and satisfaction of users with the app. 

Who can participate? 
German speakers who are aged 18 years or older, provide consent to the study and have no current or previous skin cancer.

What does the study involve? 
Participants will be recruited in the waiting room of the travel clinic of the University of Zurich (UZH Zentrum für Reisemedizin). Upon entering the waiting room, potential participants will be informed about the study and asked if they voluntarily would like to take part. Those who volunteer and fulfill all participation criteria will be informed in detail about the study and asked to provide their written consent. 

Each participant will then be allocated to one of two groups. One group will have full access to the app (including the online questionnaire and online feedback). The other group will only have access to the online questionnaire. The app will be accessed via a tablet.

Participants will then register into the SUNsitive app and either only answer the online questionnaire or answer the questionnaire and receive the online tailored advice about skin health. The feedback will be based on the answers provided by the participant in the questionnaire.  

One week later, participants in the full access group will receive a reminder email, asking them to access the SUNsitive app from home and to read the advice and feedback given on the app again. 

Two weeks after participation, both groups will again receive the questionnaires through the app, which they will have to answer again in order to see whether any changes occurred.

What are the possible benefits and risks of participating? 
SUN-sitive will contribute towards a much needed and targeted increase in awareness around sun protection, melanoma prevention, skin health and the linkages between tobacco consummation and the skin. Based on previous research, it is expected that SUN-sitive exposure will impact individual attitudes towards tanning and UV-ray exposing behavior, which is a strong predictor for actual behavior.

This trial can be categorized involves minimal risk or burden. SUNsitive is non-invasive, does not target a vulnerable population and is not expected to cause any discomfort or burden. 

Providing information on risks and dangers (which SUNsitive will do through its feedback modules) always carries a risk to cause individual concern or worry. SUN-sitive will counteract that by using clear, non-emotional language, based on facts and evidence, allowing for questions and concerns to be discussed with the healthcare provider during the consultation, and allowing for questions and concerns to be directly discussed during SUN-sitive exposure with a member of the team. Part of the preventive feedback will be an estimation of melanoma risk, based on a validated score. We will ensure that the participants are informed that this risk score is only estimated and that it strongly depends on the accuracy of the provided information.

Where is the study run from? 
University of Zurich Travel Clinic (Switzerland)

When is the study starting and how long is it expected to run for? 
April 2020 to November 2020

Who is funding the study? 
The Béatrice Ederer-Weber Foundation (Switzerland) and the University of Zurich Digital Society Initiative (Switzerland)

Who is the main contact? 
Mr Vasileios Nittas
vasileios.nittas@uzh.ch

**Trial website**

<https://sunsitive.rmis-uzh.ch/login>

Contact information

**Type**

Scientific

**Primary contact**

Prof Milo Puhan

**ORCID ID**

<https://orcid.org/0000-0003-4721-1879>

**Contact details**

Hirschengraben 84
Zurich
8001
Switzerland
+41 44 634 46 10
[miloalan.puhan@uzh.ch](mailto:miloalan.puhan@uzh.ch)

**Type**

Scientific

**Additional contact**

Dr Margot Mütsch

**ORCID ID**

<https://orcid.org/0000-0003-0620-5376>

**Contact details**

Hirschengraben 84
Zurich
8001
Switzerland
+41 44 63 44857
[margot.muetsch@uzh.ch](mailto:margot.muetsch@uzh.ch)

**Type**

Scientific

**Additional contact**

Mr Vasileios Nittas

**ORCID ID**

**Contact details**

Hirschengraben 84
Zurich
8001
Switzerland
+41 44 63 44946
[vasileios.nittas@uzh.ch](mailto:vasileios.nittas@uzh.ch)

Additional identifiers

**EudraCT number**

Nil known

**IRAS number**

**ClinicalTrials.gov number**

Nil known

**Protocol/serial number**

Nil known

Study information

**Scientific title**

Assessing the Effects of Digital Skin Health Promotion and Sun Protection Advice: The SUNsitive Project

**Acronym**

SUNsitive

**Study hypothesis**

Exposure to SUNsitive (sun protection and skin health promotion web application) will increase participant intentions to protect their skin from UV-radiation

**Ethics approval**

An ethics approval was requested by the Cantonal Ethics Committee Zurich and was waived as the research project does not fall within the scope of the Human Research Act (HRA)

**Study design**

Single-centre, two-arm randomized controlled trial

**Primary study design**

Interventional

**Secondary study design**

Randomised controlled trial

**Trial setting**

Internet

**Trial type**

Prevention

**Patient information sheet**

Not available in web format, please use contact details to request a participant information sheet

**Condition**

Sun protection, melanoma prevention, skin health promotion, including awareness of tobacco effects on skin health

**Intervention**

Current interventions as of 30/09/2020: 

Participants will be randomized, in a ratio of 1:1, into one of two arms, the intervention group or the control group, using an online research randomization software. Participants will be drawn from an online panel sample, representative of the Swiss population, pre-stratified according to age (18-44; 45-75) and gender (m; f)

The intervention group will receive access to a web-based app providing tailored sun protection, melanoma prevention, and skin health promotion advice, following the completion of a survey at recruitment (baseline). The same survey will also be completed at follow-up (after two weeks).

The control group will receive care as usual and will also answer the same surveys at baseline and two weeks and only receive generic feedback.

Both groups will complete a usability survey at 2 weeks.

_____

Previous interventions:

Participants will be randomized, in a ratio of 1:1, into one of two arms, the intervention group or the control group, using an online research randomization software.

The intervention group will receive access to a web-based app providing tailored sun protection, melanoma prevention, and skin health promotion advice, following the completion of a survey at recruitment (baseline). The same survey will also be completed at follow-up (after two weeks).

The control group will receive care as usual and will also answer the same surveys at baseline and two weeks.

**Intervention type**

Behavioural

**Phase**

**Drug names**

**Primary outcome measure**

Sun protection intentions measured by a 16-item questionnaire (adapted from Mahler, H.I.M., Kulik, J.A., Gerrard, M., & Gibbons, F. X. (2010). Effects of upward and downward social comparison information on the efficacy of an appearance-based sun protection intervention: A randomized, controlled experiment. Journal of Behavioral Medicine), at baseline and 2 weeks

**Secondary outcome measures**

1. Self-efficacy to sun protect measured by a 6-item questionnaire (adapted from Babbin, S. F., Yin, H. Q., Rossi, J. S., Redding, C. A., Paiva, A. L., & Velicer, W. F. (2015). Reducing sun exposure for prevention of skin cancers: factorial invariance and reliability of the self-efficacy scale for sun protection. Journal of skin cancer, 2015), at baseline and 2 weeks
2. Attitudes towards tanning measured by a 6-item questionnaire (adapted from Mahler, H. I., Kulik, J. A., Gerrard, M., & Gibbons, F. X. (2010). Effects of upward and downward social comparison information on the efficacy of an appearance-based sun protection intervention: a randomized, controlled experiment. Journal of Behavioral Medicine, 33(6), 496-507), at baseline and 2 weeks
3. Solarium use intentions measured by a 2-item questionnaire (adapted from Heckman, C. J., Manne, S. L., Kloss, J. D., Bass, S. B., Collins, B., & Lessin, S. R. (2011). Beliefs and intentions for skin protection and UV exposure in young adults. American journal of health behavior, 35(6), 699-711), at baseline and 2 weeks
4. Smoking status measured by a 7-item questionnaire (adapted from Etter, J. F., & Sutton, S. (2002). Assessing ‘stage of change’in current and former smokers. Addiction, 97(9), 1171-1182), at baseline and 2 weeks
5. Feasibility and Usability of the SUNsitive application measured by a questionnaire designed by the investigators at 2 weeks

**Overall trial start date**

01/03/2019

**Overall trial end date**

30/01/2021

**Reason abandoned (if study stopped)**

Eligibility

**Participant inclusion criteria**

Current exclusion criteria as of 30/09/2020: 

1. Aged ≥18 years
2. Good understanding of German 
3. Able to independently provide informed consent 
4. No previous skin cancer diagnosis
5. Resident in Switzerland

_____

Previous inclusion criteria:

1. Aged ≥18 years
2. Good understanding of German 
3. Able to independently provide informed consent

**Participant type**

Healthy volunteer

**Age group**

Adult

**Gender**

Both

**Target number of participants**

200

**Participant exclusion criteria**

1. Previous or current skin cancer diagnosis 
2. Prescription of doxycycline during travel medicine consultation (assessed post-consultation)

**Recruitment start date**

20/11/2020

**Recruitment end date**

31/12/2020

Locations

**Countries of recruitment**

Switzerland

**Trial participating centre**

**Epidemiology, Biostatistics and Prevention Institute, University of Zurich**
Hirschengraben 84
Zurich
8001
Switzerland

Sponsor information

**Organisation**

University of Zurich

**Sponsor details**

Epidemiology
Biostatistics and Prevention Institute
Hirschengraben 84
Zurich
8001
Switzerland
+41 44 634 46 11
[vasileios.nittas@uzh.ch](mailto:vasileios.nittas@uzh.ch)

**Sponsor type**

University/education

**Website**

<https://www.ebpi.uzh.ch/en.html>

**GRID**

[grid.7400.3](https://www.grid.ac/institutes/grid.7400.3)

Funders

**Funder type**

University/education

**Funder name**

Béatrice Ederer-Weber Stiftung

**Alternative name(s)**

**Funding Body Type**

**Funding Body Subtype**

**Location**

**Funder name**

Universität Zürich

**Alternative name(s)**

University of Zurich, UZH

**Funding Body Type**

government organisation

**Funding Body Subtype**

Local government

**Location**

Switzerland

Results and Publications

**Publication and dissemination plan**

Our results will be published in peer-reviewed scientific journals, in the field of digital health. 

IPD Sharing statement:
The data sharing plans for the current study are unknown and will be made available at a later date

**Intention to publish date**

01/03/2021

**Participant level data**

To be made available at a later date
