# Supplementary material for: Effectiveness of a tailored web app on sun protection intentions and its implications for skin cancer prevention: A randomized controlled trial
Source: PLOS Digit Health. 2022 May 12;1(5):e0000032. doi: 10.1371/journal.pdig.0000032 (PMC9931317; doi:10.1371/journal.pdig.0000032)
Supplement: S1 Ethics Approval — (PDF) [file pdig.0000032.s005.pdf]

Kanton Zürich
Kantonale Ethikkommission

Prof. Dr. med. Peter Meier-Abt
Präsident

Dr. med. Peter Kleist
Geschäftsführer
Stampfenbachstrasse 121
Postfach
8090 Zürich
Telefon +41 43 259 79 70
Fax +41 43 259 79 72
www.kek.zh.ch

University of Zurich
Epidemiology, Biostatistics and Prevention
Institute
Vasileios Nittas
Hirschengraben 84
8001 Zurich

08. November 2019 / mom

BASEC-Nr. Req-2019-01102
Clarification of responsibility

SUN-sitive: Assessing the Effects of Digital Melanoma Prevention and Skin Health Promotion

Dear Mr Nittas

We refer to your submission dated 06.11.2019

Your research project does not fall within the scope of the Human Research Act (HRA). Therefore, an authorization from the ethics committee is not required.

Kindly note that an invoice in the amount of CHF 100.- will be issued by the cantonal accounts department.

Sincerely yours,

Peter Kleist
